# Prioritizing cancer therapeutic genes using BioRank: A biologically-informed PageRank framework

**DOI:** 10.1016/j.csbj.2025.09.032

**Published:** 2025-10-01

**Authors:** Duc-Tinh Pham, Huu-Tam Nguyen, Van-Hai Pham, Van-Thanh Le

**Affiliations:** a School of Information and Communications Technology, Hanoi University of Industry, 298 Cau Dien street, Bac Tu Liem District, Hanoi, Viet nam; b School of Information and Communications Technology, Hanoi University of Science and Technology, Hanoi, Viet nam; c Graduate University of Science and Technology, Vietnam Academy of Science and Technology, Hanoi, Viet nam; d Cyber School, Vinh University, Nghean, Viet nam

**Keywords:** BioRank, Cancer, Drug target gene, Biomolecular networks, Gene expression information

## Abstract

The identification of therapeutic target genes constitutes a critical yet challenging aspect of cancer research, primarily due to the inherent complexities of biological systems and the heterogeneity of molecular data. This study introduces BioRank, an innovative gene prioritization methodology that extends the traditional PageRank algorithm by integrating biological insights through a custom-designed vector. This vector synthesizes differential gene expression, functional annotations (derived from GO, KEGG, and Reactome), and coexpression similarity to achieve a classification of enhanced biological significance. BioRank was validated using RNA sequencing data from The Cancer Genome Atlas (TCGA), alongside protein–protein interaction networks from HIPPIE across seven cancer datasets. Experimental results illustrate that BioRank effectively facilitates the identification and prioritization of therapeutic target genes. Comparative analysis with previous methodologies indicates that BioRank achieves superior predictive performance concerning both the number of target genes in OncoKB, as well as Recall@ and nDCG@ metrics. BioRank operates as a research instrument designed for hypothesis generation, prioritizing candidate therapeutic target genes based on a specified cancer type and standard molecular/network inputs. This empowers researchers to prioritize genes for subsequent biological validation, such as functional assays, while simultaneously retrieving known targets and identifying under-explored candidates.

## Graphical Abstract

**Figure:**
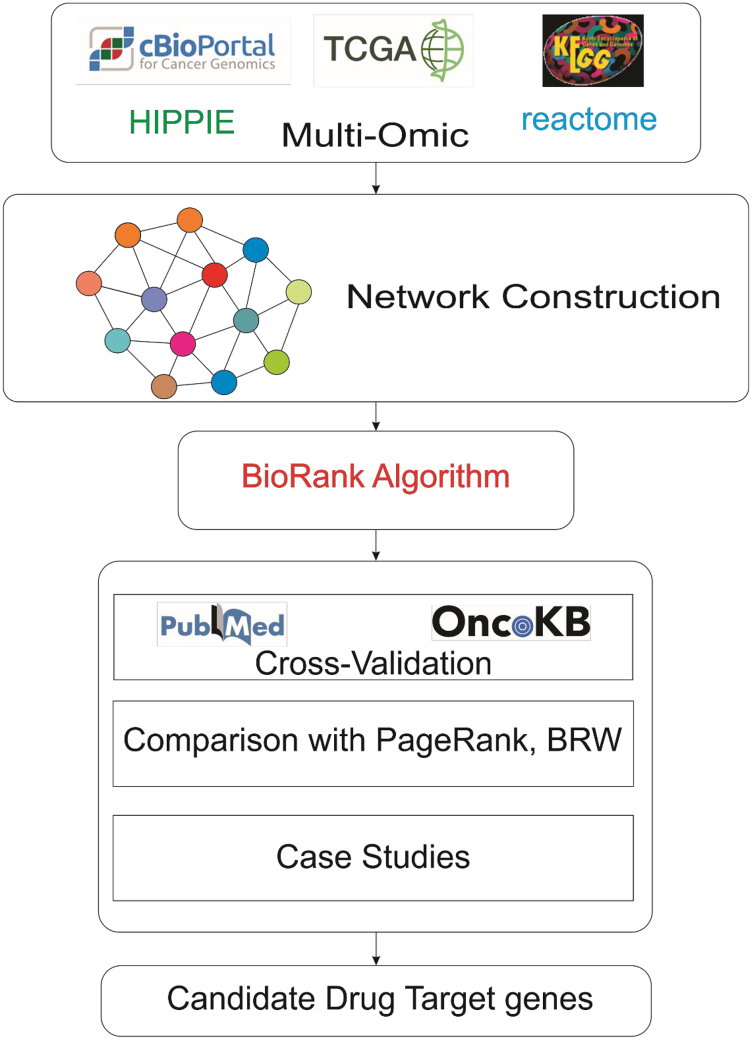

## 1 Introduction

The advent of high-throughput technologies has enabled the representation of biological data as complex networks, where nodes denote genes or proteins and edges signify their interactions [1], [2]. Employing this network paradigm, numerous computational models have been developed to assess the significance of nodes and edges. Genes or proteins that receive high rankings from these models frequently align with known disease targets, while those without prior evidence may emerge as novel candidates for experimental validation, consequently reducing the time and costs associated with wet-lab experiments.

Within the realm of network-based analytical methodologies, the PageRank algorithm [3] is employed to evaluate the prominence of individual nodes (genes/proteins within a biological network) grounded on the inherent connectivity framework of the network. Nodes (genes) that attain higher rankings are postulated to exert a substantial influence on pathological processes, thereby facilitating the identification of disease-associated genes and the exploration of potential therapeutic targets in cancer research. Nevertheless, conventional implementations of the PageRank algorithm predominantly focus on network topology, to the exclusion of critical biological attributes such as gene expression levels, functional annotations, and the biological similarities among genes [4]. Numerous studies have endeavored to address this limitation by incorporating biological information within the PageRank framework. Yet, these methodologies have frequently failed to fully exploit the synergy between interaction networks and gene-level biological features, resulting in suboptimal accuracy for the prioritization of cancer genes [5]. For instance, [6] employed a weighted PageRank algorithm to identify disease-associated genes utilizing PPI data, where edge weights represent the confidence scores of protein interactions. Although this approach improved prioritization by taking into account interaction reliability, it neglected other crucial biological data such as gene expression profiles, annotations, or pathway information, thus limiting its efficacy, particularly for complex diseases such as cancer, where the integration of multimodal data is essential. In another study, [7] compared various network propagation methods and examined multi-layer data integration by fusing both PPI and gene expression networks. Despite the performance enhancements achieved through multi-omics integration, the PageRank formulation was not tailored to specific data types or disease contexts, and pathway-specific or annotation data were not incorporated, thereby constraining the model’s ability to accurately identify disease-associated genes. Building on this research trajectory, [8] proposed a multi-layer molecular interaction network derived from heterogeneous omics data such as RNA-seq, miRNA-seq, and other gene-level features. While this approach leveraged network heterogeneity, its efficacy was heavily dependent on the quality and completeness of the input data, with incomplete or inconsistent datasets significantly impairing predictive accuracy. More recently, [9] introduced the Constrained PageRank (CPR), which integrates multiple omics layers, including RNA-seq, DNA methylation, miRNA, and somatic mutations, into a coherent biological network to enhance disease gene prediction. Despite demonstrating substantial improvements over previous models, CPR also contended with issues of missing or inconsistent input data. Additionally, the lack of pathway-specific or gene annotation integration limited its applicability to complex diseases such as cancer, where such information is crucial for precise gene ranking.

**Fig. 1:**
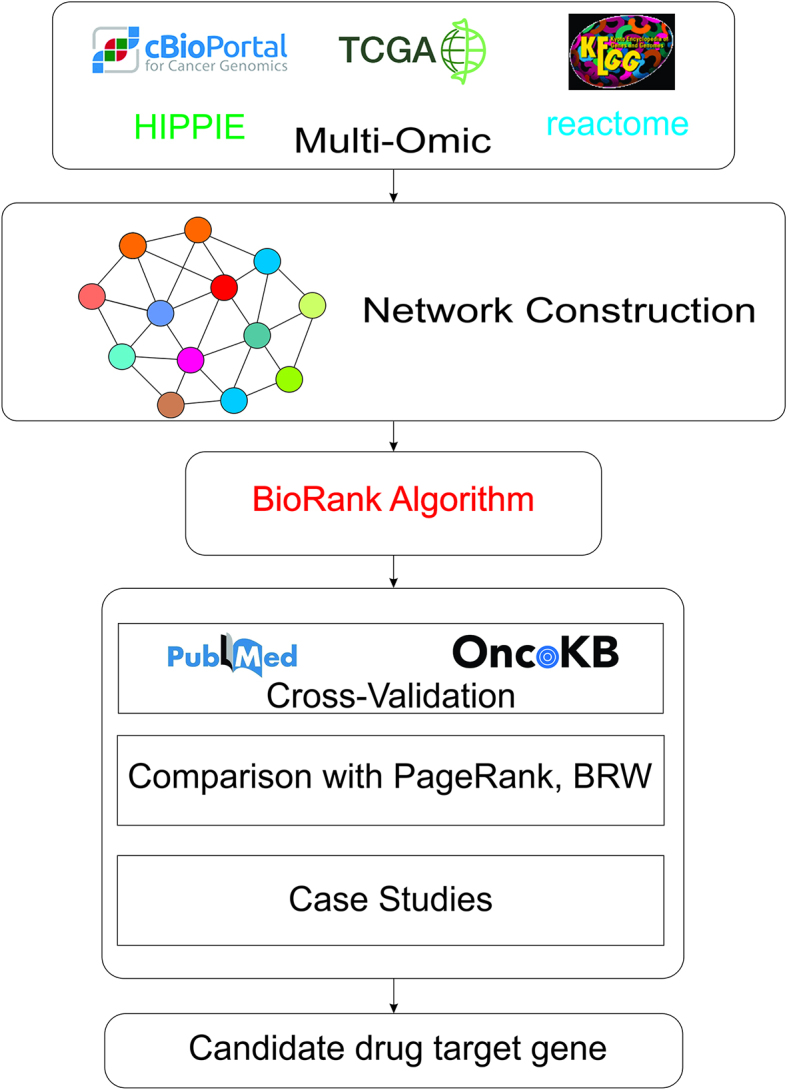
Overview of the BioRank framework for therapeutic gene prioritization.

**Fig. 2:**
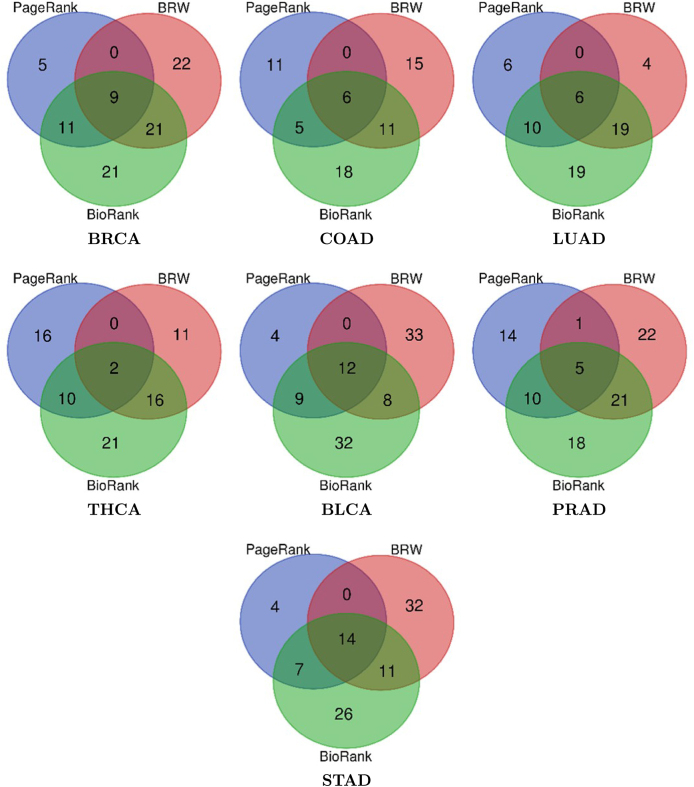
Shared and unique target genes predicted top 100 by BioRank, BRW, and PageRank across cancer datasets. A comparative visualization of the therapeutic gene predictions generated top 100 by BioRank, BRW, and PageRank across seven cancer datasets. This figure effectively demonstrates both the concordance and divergence among the three algorithms. It is noteworthy that BioRank consistently identifies the majority of known targets detected by existing methods, while also contributing a significant number of unique predictions, many of which are corroborated by PubMed evidence. This dual characteristic highlights BioRank’s capacity to balance sensitivity (recovering established cancer genes) and novelty (identifying underexplored candidates).

In this study, we introduce BioRank, a tool designed to facilitate the identification and prioritization of therapeutic target genes, reinforce the scientific basis for potential candidate genes, and propose novel candidate genes prioritized for subsequent biological experiments. BioRank represents an advancement over PageRank, demonstrating efficiency in biological data analysis due to its comprehensive strategy of integrating essential biological features of genes and proteins from diverse heterogeneous sources. Specifically, gene annotation information from Gene Ontology [10], Reactome [11], and KEGG Pathway [12], [13] is utilized to assess the functional relevance of genes to cancer. Moreover, differential gene expression analysis is employed to determine expression changes between tumor samples and controls. A personalized vector synthesized from multiple biological information sources, is employed to more accurately capture the biological role and significance of each gene within the network. Furthermore, a convex combination strategy is implemented to optimize the contributions of various data sources. These enhancements allow BioRank to surpass previous gene prioritization methods by leveraging both the network structure and the detailed biological attributes of each gene. Our findings contribute to the advancement of precision medicine [14], [15] and facilitate the reduction of sample sizes needed for assay validation in clinical settings [16] (Fig. 1, Fig. 2).

The inputs consist of TCGA RNA-seq tumor data, the HIPPIE protein-protein interaction (PPI) network, and functional annotations derived from Gene Ontology (GO), KEGG, and Reactome. Node evidence is synthesized from (i) enrichment of annotations and (ii) differential expression between tumor and control conditions, as well as network proximity, culminating in a personalized vector (q = αθ + (1 - α)ϕ). Edges are assigned weights based on functional similarity and co-expression (W = β W_1_ + (1 - β)W_2_). A personalized PageRank algorithm, incorporating a damping factor d, is employed to propagate scores within the weighted network to generate a prioritized list of candidate genes. The top predictions are evaluated against OncoKB and corroborated by PubMed, with performance assessed using Recall@ and nDCG@ metrics across seven TCGA cancer types: BRCA, COAD, LUAD, THCA, BLCA, PRAD, and STAD.

## 2 Data and method

### 2.1 Data

In the development and training of the BioRank model, four primary types of input data were employed:

**The protein–protein interaction (PPI) network** was acquired from HIPPIE version 2.2 database [17], a dedicated repository offering detailed information on human protein interactions, accompanied by confidence scores derived from diverse evidence sources. Only those interactions with confidence scores exceeding 0.7 were retained, thereby ensuring the precision and dependability of the network. The refined PPI network constituted the foundational graph for signal propagation within the enhanced PageRank algorithm.

**Gene Ontology Annotations (Ontology Graph).** Each gene can be associated with a variety of biological descriptors, including functions, biological processes, and signaling pathways. Annotation data were assimilated from three principal sources: Reactome [11], Gene Ontology (GO) [10], and KEGG pathways [12], [13]. The annotations underwent statistical filtration using Fisher’s Exact Test and False Discovery Rate (FDR) correction [18], [19] to exclude associations deemed unreliable. This procedure guarantees that only statistically significant associations are preserved for the construction of the ontology graph.

**Seed Genes.** For each cancer type, a seed gene set was developed consisting of genes verified to be linked with the respective disease. These genes were sourced from cBioPortal for Cancer Genomics [25] and subsequently refined to include solely cancer driver genes exhibiting a mutation frequency exceeding 1 %. This seed set served as the initial input for the personalized vector in the enhanced PageRank model.

**Gene expression profiles** were retrieved from the TCGA database [20], encompassing both tumor and control samples. We conducted an analysis of RNA-seq data to generate cancer-specific tumor-control gene expression matrices for each cancer type. These matrices facilitated the identification of differentially expressed genes and co-expression networks throughout the preprocessing phase.

In order to assess the performance of the model, a validation set consisting of disease-associated genes sourced from the OncoKB database [26], [27] was utilized. The OncoKB database is a curated repository of cancer-related genes, underpinned by clinical evidence and associations with FDA-approved therapeutics. This gene set functioned as the ground truth for evaluating the accuracy and effectiveness of the proposed model.

### 2.2 Node weight computation based on biological information

In the present investigation, we employed the methodology delineated in [21] to determine both node and edge weights by integrating protein–protein interaction (PPI) networks with a wide array of biological data sources. Our algorithm deviates from the traditional reliance on node degree, which is the count of interactions per gene, by also incorporating gene annotation data and gene expression levels sourced from repositories such as Gene Ontology (GO) [10] and KEGG pathways [12], [13]. In contrast to the conventional PageRank algorithm, where all nodes are initialized with uniform importance scores [22], which inadequately addresses the biological heterogeneity inherent in real-world molecular networks, BioRank initializes node scores using a composite metric derived from both gene annotations and gene expression data. This approach more accurately mirrors the functional and transcriptional significance of each gene.

#### A) annotation-based node weight computation

Assume we have l distinct biological sources, each providing gene annotations for the set of genes under study. Let S denote the seed gene set. To eliminate unreliable annotations, we apply statistical enrichment analysis using the Fisher Exact Test [18]. Subsequent correction for multiple comparisons is performed using the False Discovery Rate (FDR) approach [19], with level $P\_value<10^{-5}$.

For every source j∈{1,…,l}, define $F^{j}$ as the subset of annotations exhibiting significant enrichment in at least one gene from the seed set S. The aggregate set of reliable annotations F encompassing all sources is delineated as:

(1) $$F=\overset{l}{\underset{j=1}{⋃}}F^{j}$$

For each gene i (regardless of whether i∈S), let A(i) be the set of annotations assigned to gene i from all sources. The annotation-based biological score $\theta_{i}$ of gene i is then computed as follows:

(2) $$\theta_{i}=\left\{ \begin{array}{ll} \ell , & \text{if }i\in S\\ \sum_{j=1}^{l}\frac{\left|A(i)\cap F^{j}\right|}{\left|F^{j}\right|}, & \text{otherwise} \end{array}\right.$$

where, ℓ is a large constant introduced to ensure that known disease genes are assigned the highest possible priority during initialization. For genes not included in S, the value of $\theta_{i}$ is calculated based on the degree of overlap between the gene’s annotations and the set of reliable annotations, normalized by the size of each source.

#### B) expression-based node weight computation

Let $L\in R^{n\times m}$ denote the gene expression matrix. Here, n denotes the total number of genes, while m represents the number of patient samples. The element $l_{ij}$ represents the expression level of gene i in sample j.

To normalize the data, the Z-score for each element is computed as follows:

(3) $$z_{ij}=\frac{l_{ij}-\mu_{i}}{\sigma_{i}}$$

where $\mu_{i}$ is the mean and $\sigma_{i}$ is the standard deviation of the expression level of gene i across all samples.

Construct a binary matrix Z based on the Z-matrix, where:

(4) $${\bar{z}}_{ij}=\left\{ \begin{array}{ll} 1 & \text{if }z_{ij}>2.5\\ 0 & \text{otherwise} \end{array}\right.$$

A threshold of $z_{ij}>2.5$ was selected to ensure that only genes with expression levels substantially higher than the mean are considered as differentially expressed. This threshold is commonly used in transcriptomic analyses to reduce noise and increase the specificity for identifying target genes.

A gene g is considered differentially expressed if the number of patients in which gene g is identified as differentially expressed exceeds the mean value, calculated as follows:

(5) $$\frac{1}{m}\sum_{j=1}^{m}z_{gj}>\frac{1}{n\times m}\sum_{i=1}^{n}\sum_{j=1}^{m}z_{ij}$$

**Gene weight computation:** Use the PPI network to determine a topological vector ϕ and normalize $\phi_{i}$ for the differentially expressed genes:

(6) $$\phi_{i}=\frac{\left|N(i)\cap S\right|}{\left|N(i)\right|}+\frac{\left|N_{2}(i)\cap S\right|}{\left|N_{2}(i)\right|}$$

where N(i) denotes the set of genes that are immediate neighbors (first-order neighbors) of gene i, and $N_{2}(i)$ represents the set of genes located at a distance of two (second-order neighbors).

After obtaining the two weight vectors, we integrate them using a convex combination to construct the personalization vector q as input for the BioRank algorithm:

(7) q=αθ+(1−α)ϕ

where α∈[0;1] is a parameter that adjusts the balance of contribution of each data source.

### 2.3 Edge weight computation based on biological information

Edge weights reflect the interaction strength between genes in the PPI network.

#### A) using gene annotations

Construct a weighted transition matrix W, where $W_{ij}$ depends on the extent to which genes i and j share common annotations related to biological processes specific to the disease:

(8) DSI(i,j)=|A(i)∩A(j)∩F|

where A(i) is the set of annotations associated with the gene i; F is the set of statistically significant disease-related annotations.

Update the matrix W using the DSI function:

(9) $$W_{ij}^{1}=\left\{ \begin{array}{ll} c+DSI(i,j), & \text{if }(i,j)\in E\\ 0, & \text{otherwise} \end{array}\right.$$

where c is a positive constant used to ensure a minimum weight when no biological information is available at the ends of a PPI edge.

#### B) using co-expression information

(10) $$W_{ij}^{2}=\frac{P_{ij}}{\sum_{k\in V(i)}P_{ik}}$$

where $P_{ij}$ is the Pearson correlation coefficient [23] between genes i and j.

#### C) integration of edge weights from two sources

(11) $$W=\beta \cdot W^{1}+(1-\beta )\cdot W^{2}$$

where β∈[0;1] is a parameter that adjusts the balance of contributions from each data source.

### 2.4 Proposed enhanced PageRank algorithm for disease gene prioritization

The PageRank algorithm [22] can be used to evaluate the importance of each node based on the link structure of the network. The general formula for computing the PageRank score of any node v in the network is as follows:

(12) $$PR(v)=\frac{1-d}{N}+d\sum_{u\in B(v)}\frac{PR(u)}{L(u)}$$

where N is the total number of nodes, d is the damping factor (typically set to 0.85), B(v) is the set of nodes that link to node v, L(u) is the number of outbound links from node u, and PR(u) is the PageRank score of node u.

In this research, we present an innovative methodology aimed at accurately predicting potential therapeutic target genes in cancer through the introduction of an enhanced version of the PageRank algorithm. Our approach incorporates crucial bioinformatics features related to genes and proteins, drawing from annotations provided by Gene Ontology [10], Reactome [11], and KEGG Pathway information [12], [13], [24], to evaluate the relevance of genes to cancer. Subsequently, we conduct gene expression analysis to identify genes that are differentially expressed between disease samples and control samples. A protein–protein interaction (PPI) network [25] is employed to establish the network topology and allocate initial weights. Ultimately, the PageRank formula is augmented by incorporating a personalized vector, which accounts for the specific biological roles of individual genes.

Algorithm **Improved_PageRank for BioRank model**

The modified PageRank score for a node v is calculated as follows:

(13) $$PR(v)=(1-d)\cdot q(v)+d\sum_{u\in B(v)}\frac{PR(u)\cdot w(u,v)}{\sum_{k\in V(u)}w(u,k)}$$

where:

- w(u,v): edge weight between nodes u and v derived from co-expression or biological similarity, computed by:
  W=β⋅W1+(1−β)⋅W2
- $\sum_{k\in V(u)}w(u,k)$: total weight of outgoing edges from node u.
- q(v)=α⋅θ+(1−α)⋅ϕ: personalized vector of vertex v (vertex weight of v).

## 3 Results and discussion

### 3.1 Predicted results of cancer therapeutic target genes

In this study, we utilized the OncoKB database to benchmark our experimental results. OncoKB (Oncology Knowledge Base) is a well-established, cancer-specific database maintained by the Memorial Sloan Kettering Cancer Center (MSKCC), which provides comprehensive and curated information on cancer-related gene variants [26], [27]. Experiments were conducted on seven datasets corresponding to seven prevalent cancer types: breast carcinoma (BRCA), colorectal cancer (COAD), lung adenocarcinoma (LUAD), thyroid cancer (THCA), bladder cancer (BLCA), prostate cancer (PRAD), and stomach cancer (STAD). We evaluated the prediction performance of our algorithm based on the top 15 ranked genes using three metrics: (1) The number of matched therapeutic target genes found in the OncoKB database; (2) Recall@ quantifies the proportion of relevant (i.e., known disease-associated) genes that are successfully retrieved within the top K predictions generated by the algorithm; (3) nDCG@ (normalized discounted cumulative gain), which measures ranking accuracy with respect to the positions of listed genes in the prioritized results. The complete list of the top 100 ranked genes is available in the accompanying supplementary information file.

Table 1 provides a summary of the top 15 genes prioritized by BioRank across seven cancer types. It details, for each gene, its classification by OncoKB as either an oncogene or a tumor-suppressor gene, supported by relevant PubMed identifications (PMIDs listed in the table). Across various cancers, the majority of these top-ranked genes are established drivers, including tumor-suppressor genes such as TP53 (TSG), BRCA1 (TSG), and EP300 (TSG), alongside oncogenes like EGFR, ESR1, MYC, PIK3CA, and AKT1.

**Table 1:** Top 15 Prioritized Genes Across Seven Cancer Types by BioRank with Validation from OncoKB and PubMed.

CTNNB1, ERBB2, MAPK1, and SRC, thus indicating that BioRank emphasizes biologically credible targets at the top of its list. This is corroborated by high OncoKB validation rates among the top 15 (e.g., BRCA 93.3 %, BLCA 86.6 %, STAD 86.6 %, LUAD 80 %). In addition, entries within the top-ranked list that currently lack OncoKB tags are nonetheless recognized in literature as “potential candidate genes” (e.g., GRB2, SUMO2, RELA, TRIM28, FN1, ALB, PNP, CCR3, CDH7, SOX1, CCL18), suggesting they are plausible emerging targets. Furthermore, BioRank introduces a limited set of genes for “further studies” (PCDHA4, GPR161, CCL17) which are not yet curated but are prioritized through biology-based graph propagation, thereby presenting concrete hypotheses for experimental investigation. The rightmost column presents the run time per cancer-specific network (ranging from 334 to 4995 s), demonstrating scalability across networks with comparable numbers of nodes but varying edge structures. Collectively, these findings elucidate that BioRank effectively recovers established oncogenes and tumor-suppressors and identifies credible novel candidates for further validation.

### 3.2 Compared to other methods

To assess the predictive efficacy of BioRank, we conducted a comparative analysis of its outputs with those of two alternative methodologies: the original PageRank algorithm and the BRW algorithm [21], utilizing seven datasets related to prevalent cancer types. It is pertinent to mention that in study [21], the BRW algorithm underwent empirical comparison against four additional methods: RWR [28], DIAMOnD [29], DADA [30], and RWR-M [31]. Consequently, these four algorithms are excluded from our evaluation, as their comparative performance with BRW has been extensively documented. Experiments were executed using both the top 15 and top 100 ranked genes, scrutinizing various facets including the quantity of predicted genes, ranking quality, coverage, and the extent of concordance and reliability among the predicted outcomes.

It is important to acknowledge that all seven biological network datasets originate from the HIPPIE version 2.2 database, hence they encompass an identical number of genes (refer to Section 4.4, DE Gene and Co-expression Network, in the UserManual-BioRank for further details). Nonetheless, the number of edges varies as a consequence of the distinctive properties of the seed set correlated with each disease.

Table 2 provides an in-depth comparative analysis of three methodologies for gene prioritization, focusing on the top 15 genes: the conventional PageRank, the Biological Random Walk (BRW), and the newly introduced BioRank, evaluated across seven prominent cancer datasets. The assessment emphasizes three crucial metrics: the number of therapeutic target genes aligned with OncoKB (Match), the recall in the top 15 predictions (Recall@15), and the normalized Discounted Cumulative Gain (nDCG@15), appraising both relevance and ranking position. The results unequivocally demonstrate that BioRank consistently outperforms both PageRank and BRW in all three metrics across all datasets.

**Table 2:** Performance Comparison of PageRank, BRW, and BioRank on the top 15 genes across Cancer Datasets.

Biomolecular Networks	Properties		PageRank			BRW			BioRank		
	Nodes	Edges	Match	Recall@15	nDCG@15	Match	Recall@15	nDCG@15	Match	Recall@15	nDCG@15
BRCA	12,148	219,166	6	0.0051	0.4020	6	0.0060	0.3255	**14**	0.0119	**0.9265**
COAD	12,148	799,078	6	0.0051	0.3964	2	0.0034	0.2070	**10**	0.0085	**0.7422**
LUAD	12,148	337,686	7	0.0060	0.4477	2	0.0062	0.2106	**11**	0.0102	**0.8258**
THCA	12,148	547,306	7	0.0050	0.4404	3	0.0034	0.2113	**8**	0.0068	**0.5304**
BLCA	12,148	237,288	6	0.0051	0.4020	9	0.0074	0.7168	**13**	0.0111	**0.8829**
PRAD	12,148	607,492	7	0.0060	0.4194	6	0.0054	0.3755	**10**	0.0101	**0.7177**
STAD	12,148	271,464	6	0.0051	0.4005	10	0.0102	0.8604	**13**	0.0116	**0.8817**

## 4 Discussion

The predictive outcomes indicate that BioRank constitutes a promising tool engineered to aid in the identification and prioritization of therapeutic target genes, including TP53, ESR1, EGFR, AKT1, and MYC. It solidifies the scientific basis for potential candidate genes such as GRB2, SUMO2, RELA, TRIM28, FN1, ALB, PNP, CCR3, CDH7, SOX1, and CCL18. Additionally, it introduces novel candidate genes like PCDHA4, GPR161, and CCL17, thereby emphasizing them as priorities for ensuing biological experiments. A critical aspect contributing significantly to the enhanced predictive performance is the formulation of a tailored weighted vector that incorporates biological information derived from seed genes. Rather than utilizing a uniform vector, the incorporation of biologically relevant information facilitates signal propagation in a manner that accurately mirrors biological mechanisms, thereby enhancing the identification of functionally relevant genes. Moreover, edge weighting, representing both the reliability and biological significance of protein–protein interactions, is indispensable. By steering signal propagation more selectively and mitigating the dilution of signal through low-confidence edges, the method further hones its ability to prioritize biologically significant genes. An exhaustive analysis of the results implies that Recall@ and nDCG@ provide considerable advantages for gene ranking objectives. A high Recall@ and nDCG@ value signifies that validated target genes are prioritized toward the top of the predictive hierarchy, an essential factor in assisting researchers with the prioritization of candidate genes for subsequent biological experimentation.

For instance, within the context of the breast cancer dataset, TP53 functions as a critical tumor suppressor gene that is frequently inactivated in breast cancer. The TP53 Y220C variant constitutes a missense mutation within the DNA-binding domain, leading to destabilization of p53 by disrupting five electrostatic interactions [32], and fails to restore the transcriptional activity of wild-type TP53 in reporter assays [33]. ESR1 (estrogen receptor alpha) acts as a transcription factor that is commonly mutated in hormone-resistant metastatic breast carcinomas. ESR1 encodes ERα; upon estrogen binding, it facilitates the release of HSP90, instigates the dimerization of ERα/ERβ, and facilitates their translocation to the nucleus. Through interaction with ERE/AP-1/SP1 in conjunction with co-regulators, it governs cellular processes such as proliferation, migration, and differentiation [34], [35], [36], [37]. AKT1, an intracellular kinase, is frequently subject to mutation in various cancer types, including breast and endometrial carcinomas. The AKT1 E17K mutation, located within the pH-domain, is an activating mutation that enhances PI3K/AKT signaling and promotes oncogenic phenotypes [38], [39], [40], [41], [42]. This mutation is additionally associated with Proteus syndrome and breast cancer [43], [44].

Within the colorectal cancer (COAD) dataset, the epidermal growth factor receptor (EGFR) gene harbors a specific missense mutation, G465E, which is localized in the extracellular domain of the protein. This mutation has been identified in instances of colorectal cancer [45].

Within the LUAD dataset, the epidermal growth factor receptor (EGFR), a receptor tyrosine kinase, exhibits alterations through amplification and/or mutation in lung and brain malignancies, among others. In-frame deletions of exon 19 of the EGFR gene lead to constitutive activation of EGFR tyrosine kinase activity and render the receptor sensitive to tyrosine kinase inhibitors (TKIs), such as gefitinib, erlotinib, and afatinib, in lung adenocarcinoma [46], [47], [48]. The aforementioned drugs, afatinib, erlotinib, and gefitinib, have received FDA approval for the treatment of patients with non-small cell lung cancer that harbors EGFR exon 19 deletions.

## 5 Conclusion

In this study, we introduce BioRank, an advanced gene prioritization method that enhances the classical PageRank algorithm through the incorporation of multimodal biological data. Unlike conventional methodologies that rely solely on network topology, BioRank integrates gene-level annotations and expression profiles using a personalized vector, thereby enabling a biologically more meaningful initialization of nodes. The algorithm further refines the accuracy of rankings by weighting edges based on functional similarity and gene co-expression. The design of BioRank ensures that genes with biological relevance receive greater initial priority, and that signal propagation is guided by reliable interaction strengths. This approach enhances the identification of genes potentially involved in cancer pathogenesis. Validation across seven cancer-related datasets demonstrates BioRank’s superior performance compared to existing methods, as evidenced by higher Recall@, nDCG@ scores and a greater number of matched therapeutic targets listed in OncoKB and PubMed. Notably, BioRank effectively prioritized well-established cancer genes such as TP53, ESR1, EGFR, AKT1, and MYC at top ranks, while also identifying less-explored but potentially promising candidates such as GRB2, SUMO2, RELA, TRIM28, FN1, ALB, PNP, CCR3, CDH7, SOX1, and CCL18. Furthermore, it suggested novel genes not previously reported, such as PCDHA4, GPR161, and CCL17, thereby highlighting them as priorities for future biological investigations. These findings underscore the practical utility of integrating network structure with functional genomics to improve the accuracy and interpretability of cancer gene prioritization. The algorithm and its supporting resources are made publicly available to facilitate reproducibility and further research.

Planned future work (beyond the current scope) involves evaluating BioRank with harmonized

TCGA–GTEx resources (and explicit batch-correction pipelines) as a sensitivity analysis. Additionally, further experiments could assess the impact of the α and β values on predictive performance, as well as the specific contribution of each data source to the model’s predictive capacity. We contend that these additions will strengthen the manuscript while preserving the integrity and comparability of the results presented here.

## CRediT authorship contribution statement

**Duc-Tinh Pham:** Writing – review & editing, Writing – original draft, Supervision, Project administration, Methodology, Investigation, Funding acquisition, Formal analysis, Software, Conceptualization. **Huu-Tam Nguyen:** Writing – original draft, Visualization, Software, Resources, Data curation. **Van-Hai Pham:** Writing – review & editing, Supervision, Project administration. **Van-Thanh Le:** Visualization, Software, Resources, Funding acquisition.

## Declaration of competing interests

The authors declare that they have no known competing financial interests or personal relationships that could have appeared to influence the work reported in this paper.
